# Triazole-resistant *Aspergillus luchuensis*, an industrially important black *Aspergillus* spp. used in fermentation in East Asia, isolated from the patient with invasive pulmonary aspergillosis in China

**DOI:** 10.1080/22221751.2022.2076614

**Published:** 2022-05-26

**Authors:** Qiqi Wang, Yun Li, Yanming Li, Nir Osherov, Gustavo H. Goldman, Paul E. Verweij, Bo Zheng, Ruoyu Li, Wei Chen, Tianyu Liang, Zhe Wan, Wei Liu

**Affiliations:** a Department of Dermatology and Venerology, Peking University First Hospital, Beijing, People’s Republic of China; b National Clinical Research Center for Skin and Immune Diseases, Beijing, People’s Republic of China; c Research Center for Medical Mycology, Peking University, Beijing, People’s Republic of China; d Beijing Key Laboratory of Molecular Diagnosis on Dermatoses, Beijing, People’s Republic of China; e Institute of Clinical Pharmacology, Peking University First Hospital, Beijing, People’s Republic of China; f Department of Clinical Laboratory, Xiangya Hospital, Central South University, Changsha, People’s Republic of China; g Department of Clinical Microbiology and Immunology, Sackler School of Medicine, Tel Aviv University, Tel Aviv, Israel; h Faculdade de Ciências Farmacêuticas de Ribeirão Preto, Universidade de São Paulo, Ribeirão Preto, Brazil; i Department of Medical Microbiology, Radboud University Medical Center, Nijmegen, Netherlands; j Radboudumc – CWZ Center of Expertise for Mycology, Nijmegen, Netherlands

**Keywords:** *Aspergillus luchuensis*, fermentation in East Asia, triazole-resistance, *cyp51A* mutation, aspergillosis

## Abstract

*Aspergillus luchuensis*, an industrially important member of *Aspergillus* species belonging to section *Nigri* used in fermentation in East Asia, was isolated from an immunocompromised patient with probable invasive pulmonary aspergillosis who failed voriconazole therapy in China. This isolate showed non-wild-type susceptibility to itraconazole, voriconazole, isavuconazole, and posaconazole. A G1378A mutation in *cyp51A*, resulting in the G441S amino acid substitution, which is the homolog to G448S conferring triazole-resistance in *A. fumigatus*, was detected in the *A*. *luchuensis* isolate.

## Introduction

*Aspergillus* species (spp.) are the causative pathogens of invasive aspergillosis (IA) with considerable morbidity and mortality. Although *Aspergillus fumigatus* continues to be the most prevalent spp., other *Aspergillus* spp. such as *Aspergillus* section *Nigri* have been increasingly recognized to cause invasive disease [1]. *Aspergillus* section *Nigri* is widespread in the environment and used in industrial manufacture to produce pharmaceuticals, food ingredients, and enzymes [2]. *A. luchuensis*, a member of *Aspergillus* section *Nigri*, is widely used in food fermentation in East Asia, such as meju and nuruk in Korea, awamori in Japan, and Puerh tea in China [2]. *A. luchuensis* is often associated with otomycosis [3] and is not reported to cause IA. Here, we report the first case of probable invasive pulmonary aspergillosis (IPA) caused by *A. luchuensis* exhibiting triazole-resistance with a G441S mutation in *cyp51A* gene.

## Methods and results

**Figure 1.**
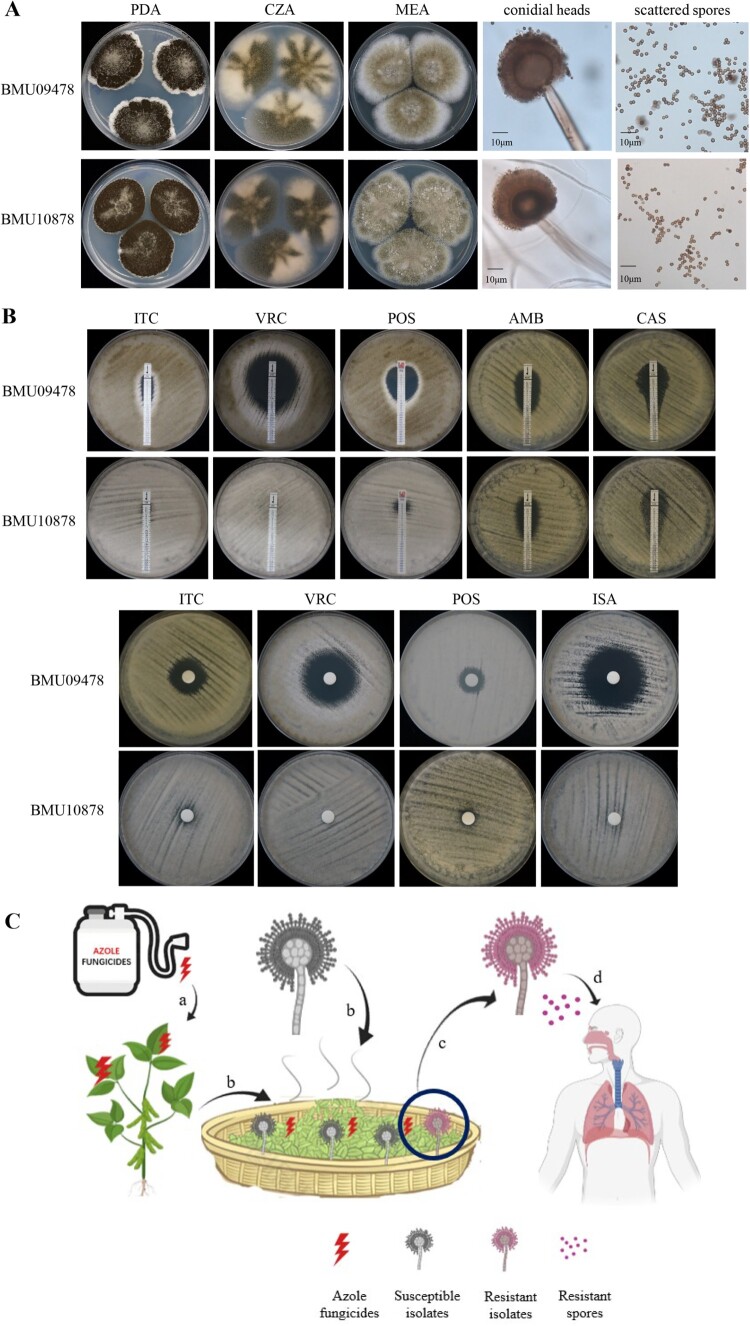
(A) Morphology of *A. luchuensis* BMU09478 and BMU10878 following 7-day-culture at 25°C. PDA: black granular colony; CZA: cottony, brown-yellow colony; MEA: velvet-like, yellow-green colony; corolla-like conidial heads with conidiogenous cells; scattered spores. (B) Antifungal susceptibilities of BMU09478 and BMU10878 to ITC, VRC, POS, AMB, CAS determined by E-test; ITC (80 μg), VRC (10 μg), POS (10 μg), ISA (80 μg) determined by disk diffusion. ITC, itraconazole; VRC, voriconazole; POS, posaconazole; ISA, isavuconazole; AMB, amphotericin B; CAS, caspofungin. (C) (a) Crops are exposed to triazole fungicides. (b) Crops applied with fungicides are further fermented with *A. luchuensis*. (c) Triazole-resistant isolates of *A. luchuensis* are selected by residues of the fungicides. (d) Triazole-resistant spores inhaled by immunocompromised patient causing IPA and failing in triazole-therapy.

A 60-year-old male patient complained of recurrent cough with bloody sputum for seven months. He also suffered from myalgia, tinnitus, and hearing loss. Chest computed tomographic (CT) scan demonstrated bilateral pulmonary masses. Anti-neutrophil cytoplasmic/proteinase-3 antibodies (c-ANCA/PR3) test was positive. Granulomatosis with polyangiitis (GPA) was diagnosed. He was initially treated with oral prednisone (1 mg/kg/d). Five months later, he developed a deteriorating cough with brown sputum. CT scan revealed bilateral pulmonary cavitary lesions. The sputum sample was culture positive for *Aspergillus* spp. Therefore, the diagnosis of IPA was suspected [4] and oral voriconazole (VRC, 200 mg twice daily) was initiated. Prednisone was continued for the GPA treatment. However, the cough with sputum persisted, and breathlessness and fever developed. A bronchoscopy was performed and bronchoalveolar lavage fluid (BALF) was culture positive for *Aspergillus* spp. which was identified by macroscopic and microscopic characteristics on potato dextrose agar (PDA), Czapek agar (CZA) and malt extract agar (MEA) at 25°C for 7 days (Figure 1(A)), and by sequencing of β-tubulin and calmodulin genes (GenBank accession number: MZ028459, MZ028460). The isolate was identified as *A. luchuensis* (ID number BMU10878). Because of the poor response to VRC, antifungal susceptibility of BMU10878 to itraconazole (ITC), VRC, posaconazole (POS), isavuconazole (ISA), amphotericin B (AMB), caspofungin (CAS) was determined by the broth microdilution method according to the Clinical and Laboratory Standards Institute (CLSI) M38-A3 document [5]. Another isolate of *A. luchuensis*, BMU09478 was included for comparison. According to the epidemiological cutoff values (ECVs) for *A. niger* [6], as no clinical breakpoints (CBPs) and ECVs were established for *A. luchuensis*, BMU10878 showed non-wide-type susceptibility to ITC, VRC, ISA (all MICs > 16 μg/mL), and POS (MIC = 1 μg/mL), while the MICs of ITC, VRC, POS, ISA against *A. luchuensis* BMU09478 control isolate were 0.25 μg/mL. The MICs of AMB against both isolates were 2 μg/mL. Additionally, E-test and disk diffusion were performed and the results (Figure 1(B)) were consistent with those observed by the broth microdilution method. BALF galactomannan (GM) was 15.23 and serum GM was 8.83 (Platelia Aspergillus EIA, BioRad), which enables the classification of this infection as probable IPA [4]. The patient was subsequently admitted to the respiratory intensive care unit (ICU) because of respiratory deterioration. Intravenous liposomal-AMB (5 mg/kg/d) was given, replacing VRC, since liposomal-AMB is recommended for the treatment of triazole-resistant IA [1]. The clinical condition of the patient was improved and he could be discharged from the hospital after one month, with AMB-treatment completed for 6 weeks.

Since triazole-resistance in *Aspergillus* spp. is mainly conferred by mutations in the *cyp51A* gene encoding sterol 14-α demethylase, the open reading frame (ORF) and the promoter region of *cyp51A* gene in both isolates were amplified and sequenced (primers: F: 5’-TGTCGTTCCATCGTCATTGC-3’, R: 5’-CGTCTCTCCCAGCCTACAAT-3’), and aligned against that of *A. luchuensis* RIB2601 (GenBank No.: AP024441.1). A G1378A mutation in the ORF resulting in a G441S substitution with intact promoter region was detected in BMU10878, while that of BMU09478 was intact (GenBank No.: MZ028461, MW813967).

The Cyp51A amino acid sequences alignment between *A. luchuensis* and *A. fumigatus* revealed that the residue G441 in *A. luchuensis* was homologous to G448 in *A. fumigatus*, a key residue in the heme-binding region [7]. In *A. fumigatus*, G448S substitution was proven to confer triazole-resistance by gene replacement and was associated with treatment failure in an animal model [7]. Hence, triazole-resistance in BMU10878 may result from the G441S substitution in Cyp51A of *A. luchuensis*.

## Discussion

Triazoles are the main antifungals used in agriculture and clinical settings, and triazole-resistance is emerging and spreading worldwide [1]. In *A. fumigatus*, triazole-resistance resulting from *cyp51A* mutations is generally acquired via triazole-therapy in the clinical setting and use of triazole fungicides in the environment [8,9]. The latter more commonly involves tandem repeat (TR) integrations in the *cyp51A* promoter in combination with mutations in *cyp51A* and has been recovered from plant waste stockpiles in the Netherlands [10] and strawberry fields in China [11]. In *A. fumigatus*, point mutations in *cyp51A* such as G448S are commonly acquired in patients receiving long-term triazole therapy [12], but have also been reported in resistant isolated recovered from the environment [9] and proven to be induced by triazole fungicides [8]. Likewise, we hence presume that acquisition of triazole-resistance by the G441S mutation in *A. luchuensis* was also generated in the triazole-treated patient, since the resistant isolate was cultured from a patient with cavitary lung lesions during VRC therapy. Unfortunately, the first *Aspergillus* isolate was not stored and thus not available for antifungal susceptibility testing. Hence, we cannot rule out the possibility that the mutation was acquired in the environment, since *A. luchuensis* is widely used in fermentation and can be exposed to agricultural azoles during growth and storage of the fermentation products. The resulting spores with triazole-resistance could be inhaled by immunocompromised patients to cause IPA with failure of triazole-therapy (Figure 1(C)). For IPA caused by those isolates of *Aspergillus* spp. with triazole-resistance, liposomal-AMB has strongly been recommended [1]. And the case in this report has also confirmed that liposomal-AMB is an effective alternative for the treatment of triazole-resistant IPA.

In conclusion, triazole fungicides being applied to fermentable crops may be a potential driver of triazole-resistance in industrial *Aspergillus* spp. used for fermentation. Testing of these and other environmental isolates could help to confirm an environmental route of resistance selection. If confirmed, our observation would provide evidence for fungicide resistance selection beyond *A. fumigatus*, with implications for antifungal stewardship both in environmental and clinical use of triazole compounds.
